# Practical Cooling Strategies During Continuous Exercise in Hot Environments: A Systematic Review and Meta-Analysis

**DOI:** 10.1007/s40279-016-0592-z

**Published:** 2016-08-01

**Authors:** Alan Ruddock, Brent Robbins, Garry Tew, Liam Bourke, Alison Purvis

**Affiliations:** 1 Centre for Sport and Exercise Science, Sheffield Hallam University, A016 Collegiate Hall, Sheffield, S10 2BP UK; 2 Department of Sport, Exercise and Rehabilitation, Northumbria University, Room 244 Northumberland Building, Newcastle upon Tyne, NE1 8ST UK; 3 Centre for Sport and Exercise Science, Sheffield Hallam University, A121 Collegiate Hall, Sheffield, S10 2BP UK; 4 Faculty of Health and Wellbeing, Sheffield Hallam University, F616 Robert Winston Building, Sheffield, S10 2BP UK

## Abstract

**Background:**

Performing exercise in thermally stressful environments impairs exercise capacity and performance. Cooling during exercise has the potential to attenuate detrimental increases in body temperature and improve exercise capacity and performance.

**Objective:**

The objective of this review was to assess the effectiveness of practical cooling strategies applied during continuous exercise in hot environments on body temperature, heart rate, whole body sweat production, rating of perceived exertion (RPE), thermal perception and exercise performance.

**Methods:**

Electronic database searches of MEDLINE, SPORTDiscus, Scopus and Physiotherapy Evidence Database (PEDro) were conducted using medical subject headings, indexing terms and keywords. Studies were eligible if participants were defined as ‘healthy’, the exercise task was conducted in an environment ≥25 °C, it used a cooling strategy that would be practical for athletes to use during competition, cooling was applied during a self-paced or fixed-intensity trial, participants exercised continuously, and the study was a randomised controlled trial with the comparator either a thermoneutral equivalent or no cooling. Data for experimental and comparator groups were meta-analysed and expressed as a standardised mean difference and 95 % confidence interval.

**Results:**

Fourteen studies including 135 participants met the eligibility criteria. Confidence intervals for meta-analysed data included beneficial and detrimental effects for cooling during exercise on core temperature, mean skin temperature, heart rate and sweat production during fixed-intensity exercise. Cooling benefited RPE and thermal perception during fixed-intensity exercise and improved self-paced exercise performance.

**Conclusion:**

Cooling during fixed-intensity exercise, particularly before a self-paced exercise trial, improves endurance performance in hot environments by benefiting RPE and thermal perception, but does not appear to attenuate increases in body temperature.

## Key Points

**Table:** 

Meta-analyses revealed unclear effects for cooling during exercise on mean core temperature, end-exercise core temperature, mean skin temperature, mean heart rate, whole body sweat production and stand-alone self-paced performance.
Cooling during exercise was beneficial for self-paced exercise performance when cooling was applied during a period of fixed-intensity exercise before the trial.
Rating of perceived exertion and thermal perception were also improved and are likely mediators of performance during continuous exercise.

## Introduction

Sporting events are frequently scheduled in hot and humid environments (e.g. stages of the Tour de France, summer-month marathons); thus evidence-based practical strategies that can alleviate increases in body temperature, reduce the risk of heat illness and improve performance are of interest to scientists and applied practitioners. During cellular respiration, heat produced in active muscle is transferred by conduction and convection to blood and surrounding tissues [1, 2]. At the skin surface, heat must be transferred via dry and evaporative mechanisms to the environment to limit heat storage and increases in body temperature. When the rate of metabolic heat production exceeds the rate of external heat transfer [1], which frequently occurs when exercise is combined with high ambient temperature (>25 °C) and water vapour pressure, heat is stored and body temperature increases.

Performing exercise in thermally stressful environments increases the risk of exercise-associated muscle cramps, heat syncope, exhaustion, heat injury and exertional heat stroke [3] and also impairs exercise capacity and performance [4–11]. During fixed-intensity exercise, the attainment of high brain [12] and body temperature [5] and subsequent cardiovascular demands limit exercise capability [13]. In contrast, during self-paced exercise performance, external force production is limited so that the task can be completed, possibly mediated through rating of perceived exertion (RPE) [2], skin temperature [14, 15], skin wettedness [16] and thermal sensation [2, 17].

Cooling the body has been the subject of several reviews [18–22], two of which [18, 19] investigated the effects of cooling during exercise and suggested that cooling improved endurance performance in the heat. Siegel and Laursen [21], however, noted that most cooling methods are largely impractical for use during competition, a view supported by Tyler et al. [19], who recognised that some cooling methods would be unsuitable for use during performance because of their mass, potential for irritability, and sports regulations. Nevertheless, previous reviews [18, 19] included strategies that would be impractical to use during continuous exercise performance (e.g. heavy ice vests, mechanically circulated cool water and fan-assisted head cooling); although these might be useful during intermittent exercise, care needs to be taken when contextualising analyses.

Tyler et al. [19] noted that the largest improvements in performance were observed when core temperature and heart rate were both reduced by cooling, but acknowledged that further research is required to ascertain the mechanisms that explain these performance improvements. This mechanistic underpinning of performance has been identified as an important step in translational physiology [23].

Accordingly, the primary research questions were:

- In healthy participants, does practical cooling during continuous exercise in a hot environment, attenuate mean and final core temperature responses compared with a thermoneutral or no cooling condition in randomised cross-over trials?
- In healthy participants, does practical cooling during continuous exercise in a hot environment, improve self-paced endurance performance and exercise capacity compared with a thermoneutral or no cooling condition in randomised cross-over trials?

Secondary research questions sought to investigate the effects of cooling during exercise on mean skin temperature, heart rate, whole body sweat production, RPE and thermal perception, all of which have been implicated in the regulation of performance during exercise in the heat.

## Methods

This review was conducted in accordance with the Preferred Reporting Items for Systematic Reviews and Meta-Analyses (PRISMA) statement [24].

### Inclusion and Exclusion Criteria

Studies were included if:

- The participants were defined as ‘healthy’ or ‘able-bodied’ and without disability or disease that influenced exercise capability or thermoregulation.
- The study was conducted in an environment where the air temperature was ≥25 °C.
- The study used a cooling strategy that would be practical for athletes to apply during competitive performance.
- Cooling was applied during a self-paced performance trial or fixed-intensity task.
- The participants exercised continuously.
- The study was a randomised controlled trial with the comparator either being administered at a temperature from 30 to 40 °C or a no cooling trial.
- The study assessed core body temperature, skin temperature, heart rate, sweat loss, RPE, thermal comfort/perception, exercise capacity or self-paced performance as an outcome.
- The study was original research published in the English language in peer-reviewed journals (including ahead of press/online first).

We did not exclude studies based upon exercise mode or sex, but did exclude studies that were designed exclusively to investigate fluid balance. We received no funding for translation services, so only research published in the English language was included within the review.

### Information Sources and Search Strategy

Initial electronic database searches were performed up to 10 March 2015 using MEDLINE, SPORTDiscus, Scopus and Physiotherapy Evidence Database (PEDro). The electronic database search was updated on 17 May 2016. Medical subject headings (MeSH), database indexing terms, keywords and Boolean operators (AND/OR) were used in the search strategy. Terms were grouped into themes related to cooling, exercise and body temperature regulation. For SPORTDiscus, search terms included ‘cool*’, ‘cold*’, ‘cold temperature’, ‘cryotherapy’, ‘exercise’, ‘physical fitness’, ‘exercise therapy’, ‘physical exertion’, ‘sports’, ‘exercise movement techniques’, ‘core temperature’, ‘rectal or oesophageal or esophageal or intest* or tympanic AND temperature’, ‘body temperature’, ‘body temperature regulation’, ‘thermosen*’, ‘thermor*’, ‘hypothermia’, ‘hyperthermia’. All searches were conducted by the same author (AR). Search results were collated using Endnote software (Thomson Reuters, New York), and duplicates were removed. The title and abstract of the remaining studies were screened for relevance (AR). Full texts of potentially appropriate studies were obtained and independently assessed for eligibility by two authors (AR/BR) according to the inclusion criteria. Reference lists and citations (via Google Scholar search) of manuscripts and relevant review articles were examined for potentially eligible studies (AR).

### Data Extraction Process

Study characteristics including sample size, age, body mass, stature, aerobic capacity, health status, exercise mode, intensity of exercise, duration of exercise, ambient temperature and humidity, air/wind speed and description of the intervention were extracted for selected studies (AR). Means and standard deviations of the primary (core temperature and self-paced performance) and secondary outcomes (mean skin temperature, heart rate, whole body sweat production, RPE and thermal perception) for experimental and comparator groups were extracted (AR). When relevant data were not reported in the text, they were extracted from figures using GetGraph Graph Digitizer (http://www.getdata-graph-digitizer.com/index.php) by one author (AR). Validity of data extraction was verified by another author (BR). When there was reference to but no pertinent data available from the manuscript, the authors were contacted (AR). Reviewers (AR/BR) were not blinded to authors or institutions at any stage of the selection or data collection process.

### Data Items

Core temperature ( °C) was a primary outcome and defined as an assessment at either rectal, intestinal, oesophageal or tympanic sites. Mean skin temperature ( °C) was defined as at least a four-site weighted assessment using skin surface thermometry. Heart rate (beats·min^−1^) was defined as an assessment using electrocardiography or short-range telemetry. Whole body sweat loss (l) was defined as the difference in body mass pre to post assessment, taking into account fluid ingestion and urine output. RPE and whole body thermal perception were defined as choices made by participants from a perception scale. Exercise capacity tests were defined as those that had a fixed external or internal intensity applied until volitional exhaustion. Self-paced exercise performance was defined as tests whereby the participant was free to choose external intensity over a pre-determined duration, distance or set amount of external mechanical work.

### Risk of Bias in Individual Studies

Risk of bias was assessed using the 6-point Cochrane Risk of Bias assessment tool [25]. Two authors (AR/BR) independently assessed risk of bias. Appraisal of study quality was performed according to subject expertise (led by AR) and guided by the risk of bias assessment tool.

### Summary Measures

The mean and standard deviation of participant physical characteristics, health status, intensity and duration of exercise and exercise mode were used to subjectively determine methodological heterogeneity prior to meta-analysis (AR/BR). Data for experimental and comparator groups were analysed using Cochrane Collaboration’s Review Manager 5.3 (Cochrane IMS, Melbourne, Australia). Data were expressed as a standardised mean difference (adjusted Hedges’ *g*) and 95 % confidence interval (CI). If the 95 % CI included zero, we concluded there was no effect. Statistical heterogeneity was assessed using the *I* ^2^ statistic to determine the percentage of the variability in effect estimates due to heterogeneity rather than sampling error (chance). Pooled intervention effect estimates and 95 % CIs were calculated as a weighted average of the standardised mean difference estimated in individual studies. When *I* ^2^ exceeded 40 % (moderate heterogeneity), a random-effects model was used to calculate the pooled intervention effect; otherwise it was calculated using fixed-effect inverse variance. We performed a sensitivity analysis on self-paced performance trials after we found that studies had either (1) performed fixed-intensity exercise before performance trials [e.g. 60 % maximum aerobic capacity ($\dot{V}$O_2max_) for 60 min followed immediately by a time-trial] or (2) used self-paced performance trials only. Exercise to exhaustion, however, was considered to have large methodological heterogeneity for intensity and duration of exercise and was not meta-analysed.

## Results

### Participants and Included Studies

**Fig. 1:**
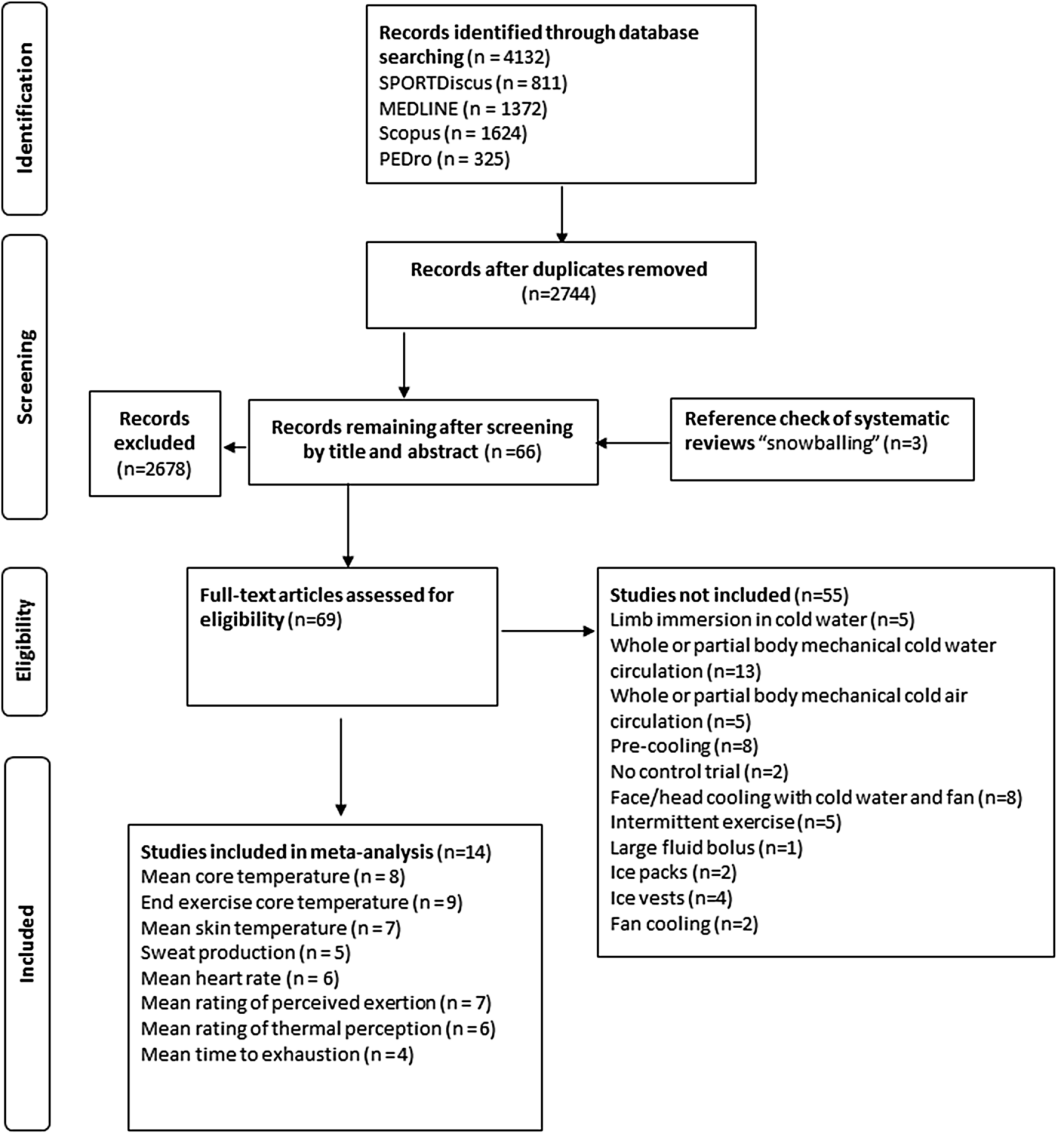
PRISMA flow diagram. *PEDro* Physiotherapy Evidence Database, *PRISMA* Preferred Reporting Items for Systematic Reviews and Meta-Analyses

Figure 1 details the PRISMA [24] flow chart. Participant characteristics are detailed in Table 1 and study details in Table 2. Mean fixed intensity was 63 ± 7 % of $\dot{V}$O_2max_, and mean duration of exercise was 74 ± 23 min, consisting of running (*n* = 7 studies) and cycling (*n* = 7) as modes of exercise. Mean ambient temperature, relative humidity and wind speed were 31 ± 2 °C, 52 ± 17 % and 2.9 ± 3.5 m·s^−1^, respectively. Participant characteristics, intensity, duration and mode of exercise and environmental conditions were considered to have small between-study methodological heterogeneity; thus a meta-analysis was performed on outcomes assessed using fixed-intensity exercise. Self-paced performance trials consisted of cycling (*n* = 4 studies) and running (*n* = 2); the mean duration of these trials was 32.6 ± 36.3 min (range from 15 to 97.4 min).

**Table 1:** Characteristics of participants in included studies

Study	Number of participants	Age (years)	Body mass (kg)	Stature (cm)	$\dot{V}$O_2max_ (ml·kg^−1^·min^−1^)	Characterisation
Burdon et al. (2010) [26]	7	33 ± 6	81.1 ± 11.1	183 ± 9	59.4 ± 6.6	Non-acclimated males
Burdon et al. (2013) [27]	10	30 ± 7	75.1 ± 9.4		61.8 ± 5.6	Healthy, naturally acclimatised male endurance cyclists
Burdon et al. (2015) [28]	10	30 ± 7	75.1 ± 9.4	175 ± 7	61.8 ± 5.6	Healthy, naturally acclimatised male endurance cyclists and triathletes
Lee et al. (2008) [29]	8	27 ± 4	70.9 ± 7.9	174 ± 5	53.8 ± 6.2	Non-heat acclimated males
Lee et al. (2014) [31]	12	24 ± 2	61.6 ± 8.1	172 ± 5	59.4 ± 5.3	Healthy males
Minniti et al. (2011) [38]	8	25 ± 5	77.4 ± 5.6	181 ± 8	53.7 ± 4.7	Healthy males
Morris et al. (2015) [32]	9	25 ± 5	75.9 ± 12.2	177 ± 7	50.9 ± 8.5	Healthy males
Scheadler et al. (2013) [33]	12	23 ± 4	76.1 ± 8.7	179 ± 6	53.8 ± 5.2	Healthy males
Schulze et al. (2015) [30]	7	33 ± 8	73.1 ± 3.3	179 ± 5	61.7 ± 3.0	Well trained male triathletes
Tyler et al. (2010) [36]^a^	9	25 ± 4	76.5 ± 5.9	181 ± 7	54.2 ± 4.6	Healthy males
Tyler et al. (2010) [36]^a^	8	25 ± 3	75.5 ± 7.0	180 ± 5	54.9 ± 3.1	Healthy males
Tyler and Sunderland (2011) [34]	8	26 ± 2	77 ± 6.2	177 ± 6	56.2 ± 9.2	Endurance trained athletes
Tyler and Sunderland (2011) [35]	7	25 ± 2	75.3 ± 8.4	179 ± 5	55.3 ± 3.6	Healthy males
Bulbulian et al. (1999) [39]	10	27 ± 6	80.5 ± 6.7	181 ± 4	38.6 ± 6.3	Healthy active males
Carvalho et al. (2014) [43]	10	25 ± 6	69 ± 2.7	170 ± 10	67.2 ± 1.8	Well trained male athletes (cyclists, mountain bikers, triathletes)

Data presented as mean ± SD

*SD* standard deviation, $\dot{V}$ *O*_*2max*_ maximum aerobic capacity

^a^ Two-part experiment

**Table 2:** Details of included studies that used fixed-intensity exercise

Study	Exercise mode	Intensity (% $\dot{V}$O_2max_)	Duration (min)	Ambient temperature ( °C)	Relative humidity (%)	Air speed (m/s)	Intervention
Burdon et al. (2010) [26]	Cycling	65	90	28.0	70.0	1.0	2.3 ml·kg^−1^ (185 ml) every 10 min of 4 °C 7.4 % carbohydrate-electrolyte solution. 37 °C 7.4 % carbohydrate-electrolyte solution. 37 °C 7.4 % carbohydrate-electrolyte solution + 30 ml ice slurry (−1 °C) every 5 min
Burdon et al. (2013) [27]	Cycling	62	90	32.1	40.0	1.0	260 ± 38 g of 7.4 % carbohydrate-electrolyte solution every 10 min as either ice slurry (−1 °C) or thermoneutral (37 °C)
Burdon et al. (2015) [28]	Cycling	62	90	32.0	40.0	1.0	260 ± 38 g of 7.4 % carbohydrate-electrolyte solution every 10 min as either ice slurry (−1 °C) or thermoneutral (37 °C)
Lee et al. (2008) [29]	Cycling	50	90	25.3	60.0		400 ml of 10 or 37 °C water ingested at 30, 45, 60 and 75 min of exercise
Lee et al. (2014) [31]	Running	70	75	30.2	71.0		155-g neck cooling collar worn throughout. No neck cooling collar
Minniti et al. (2011) [38]	Running	60	75	30.4	53.0		155-g neck cooling collar worn throughout. Uncooled collar
Morris et al. (2015) [32]	Cycling	55	75	33.5	23.7	2.25	3.2 mg·kg^−1^ (240 ml) or 37 °C of ice slurry ingested at 15-min intervals for first 45 min of exercise
Scheadler et al. (2013) [33]	Running	75	53	30.0	50.0		Refrigerated gel pack on single palm. No refrigerated gel pack
Schulze et al. (2015) [30]	Cycling		60	30.0	80.0	9.1	Ad libitum ingestion of either carbohydrate-electrolyte solution ice slurry (−1 °C) or thermoneutral beverage (30 °C)
Tyler et al. (2010) [36]	Running	60	75	30.4	53.0		155-g neck cooling collar worn throughout. Uncooled collar
Tyler and Sunderland (2011) [34]	Running	70	41	32.2	53.0		155-g neck cooling collar worn throughout. No cooling collar
Tyler and Sunderland (2011) [35]	Running	60	75	30.4	53		155-g neck cooling collar worn throughout. Neck cooling collar replaced at 30 and 60 min. No cooling collar
Bulbulian et al. (1999) [39]	Cycling	60	30	30	25	8.9	Headband and neck cooling collar soaked in ice water. No cooling

$\dot{V}$ *O*_*2max*_ maximum aerobic capacity

### Mean and End-Exercise Core Temperature

**Fig. 2:**
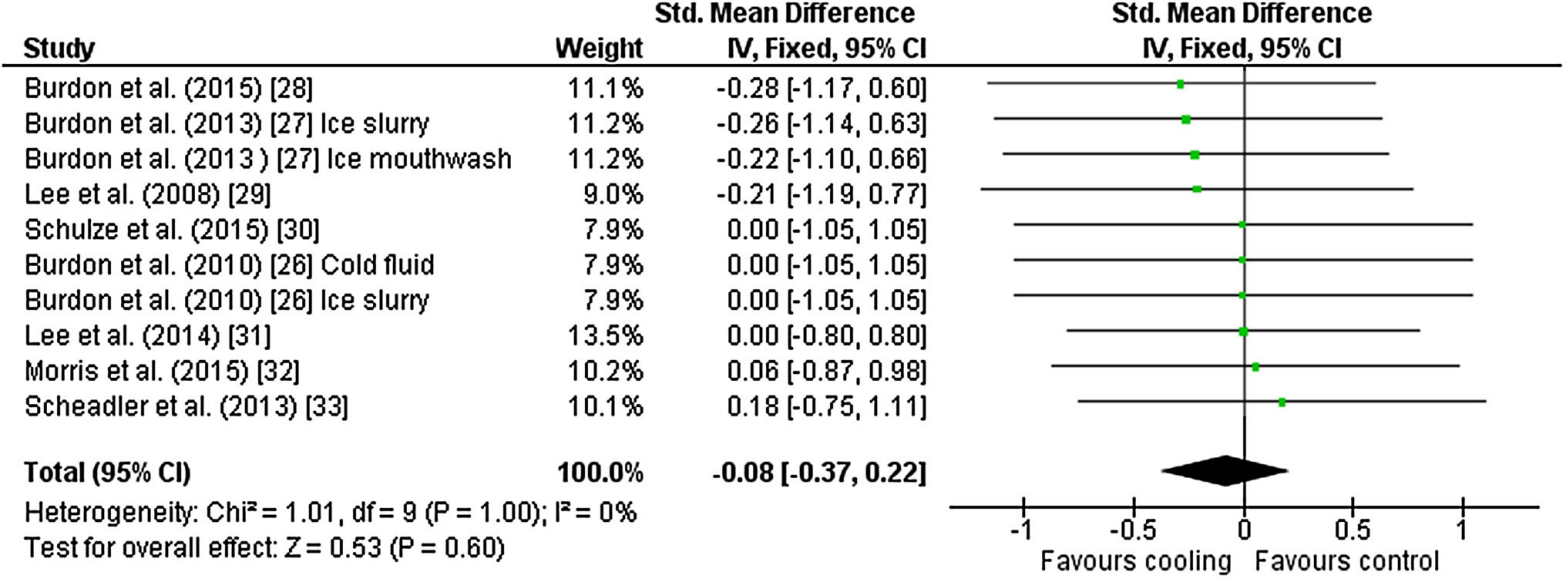
Intervention effect estimates, 95 % CIs and weighted average of the Std for mean core temperature. *CI* confidence interval, *IV* inverse variance, *SD* standard deviation, *Std* standardised mean difference

Ten outcomes from eight studies [26–33] were included in the meta-analysis for mean core temperature. Six studies used rectal temperature to assess core temperature and two studies used gastrointestinal pills. Studies used ice slurry (*n* = 5), cold fluid (*n* = 2), ice slurry mouthwash (*n* = 1), neck cooling (*n* = 1) and palm cold pack (*n* = 1) as interventions. There was no effect of cooling on mean core temperature during exercise [Hedges’ *g* = − 0.08 (95 % CI −0.37 to 0.22)] (Fig. 2).

**Fig. 3:**
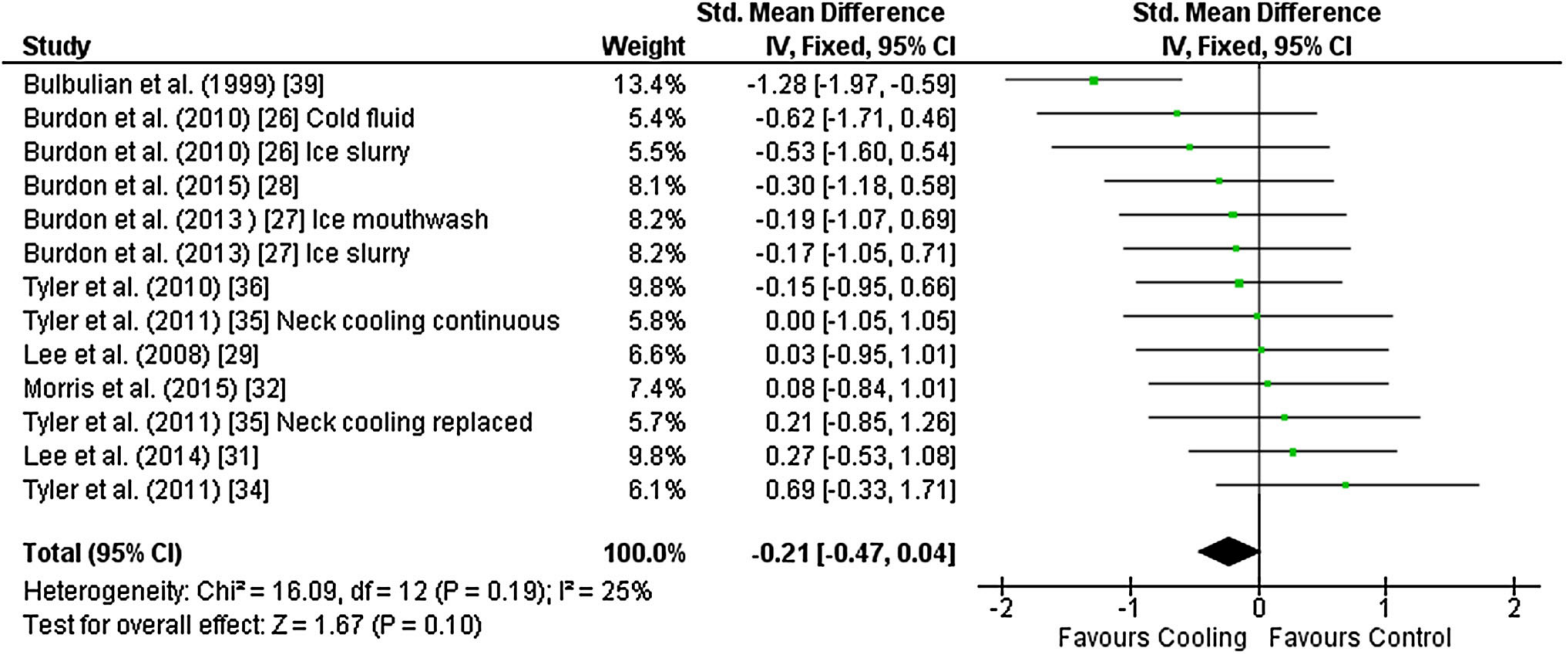
Intervention effect estimates, 95 % CIs and weighted average of the Std for end-exercise core temperature. *CI* confidence interval, *IV* inverse variance, *SD* standard deviation, *Std* standardised mean difference

Thirteen outcomes from ten studies [26–29, 31, 32, 34–36, 39] were included in the meta-analysis for end-exercise core temperature. Nine studies used rectal temperature to assess core temperature and one study used gastrointestinal pills. Studies used neck cooling (*n* = 5), ice slurry (*n* = 4), cold fluid (*n* = 2), ice slurry mouthwash and head/neck cooling (both *n* = 1) as interventions. There was no effect of cooling on core temperature at the end of exercise [Hedges’ *g* = − 0.21 (95 % CI −0.47 to 0.04)] (Fig. 3).

### Mean Skin Temperature

**Fig. 4:**
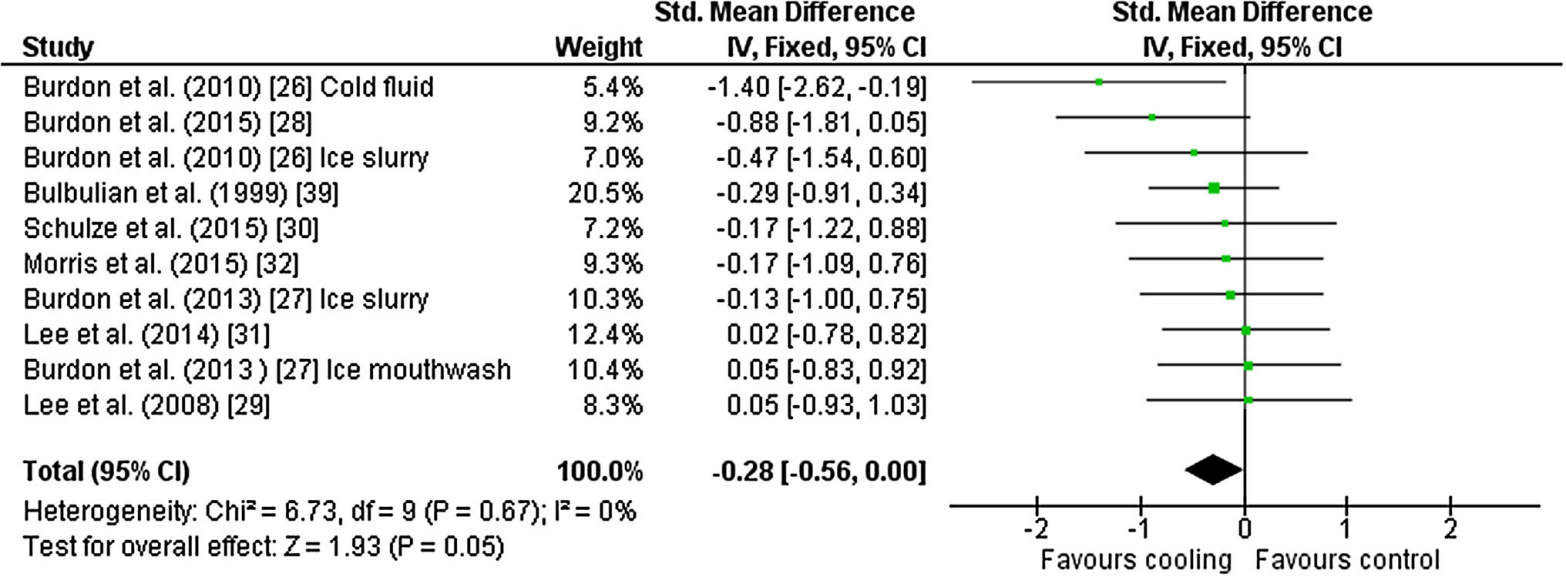
Intervention effect estimates, 95 % CIs and weighted average of the Std for mean skin temperature. *CI* confidence interval, *IV* inverse variance, *SD* standard deviation, *Std* standardised mean difference

Ten outcomes from eight studies [26–32, 39] were included in the meta-analysis for mean skin temperature. All assessments were made using weighted four-site mean calculation [37] via skin surface thermometry. Studies used ice slurry (*n* = 5), cold fluid (*n* = 2), ice slurry mouthwash (*n* = 1), neck cooling and head/neck cooling (both *n* = 1) as interventions. There was no effect of cooling on mean skin temperature [Hedges’ *g* = − 0.28 (95 % CI −0.56 to 0.00)] (Fig. 4).

### Mean Heart Rate

**Fig. 5:**
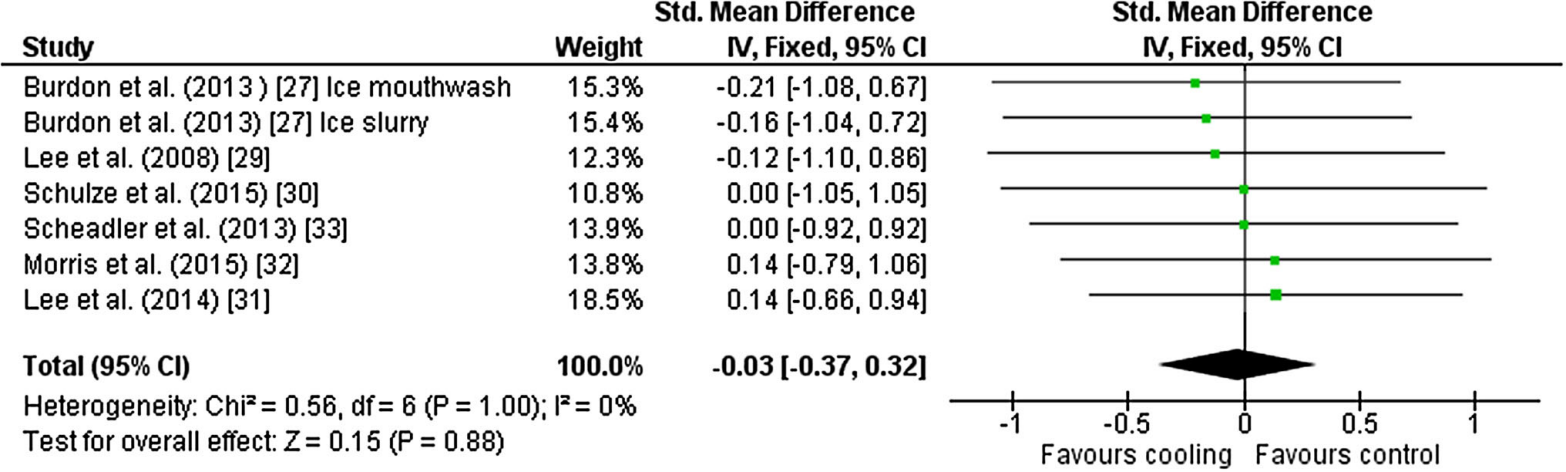
Intervention effect estimates, 95 % CIs and weighted average of the Std for mean heart rate. *CI* confidence interval, *IV* inverse variance, *SD* standard deviation, *Std* standardised mean difference

Seven outcomes from six studies [27, 29–33] were included in the meta-analysis for mean heart rate. Studies used ice slurry (*n* = 3), cold fluid, palm cold pack, neck cooling and ice slurry mouthwash (all *n* = 1) as interventions. There was no effect of cooling on mean heart rate [Hedges’ *g* = − 0.03 (95 % CI −0.37 to 0.32)] (Fig. 5).

### Rating of Perceived Exertion and Whole Body Thermal Perception

**Fig. 6:**
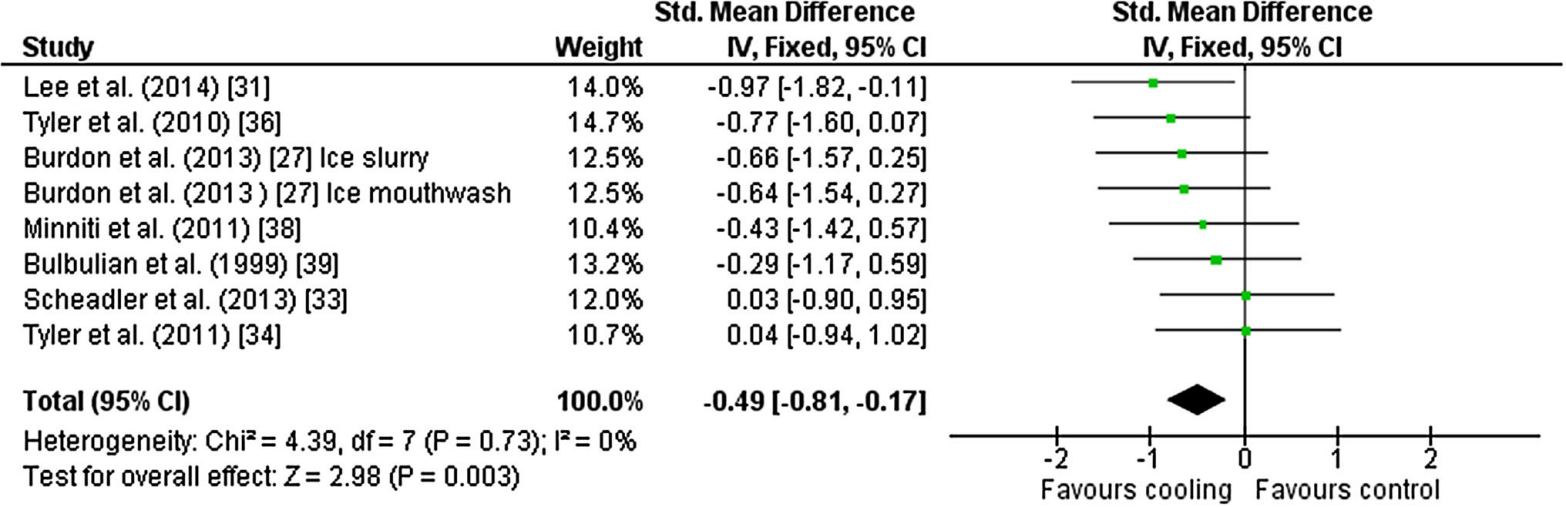
Intervention effect estimates, 95 % CIs and weighted average of the Std for mean rating of perceived exertion. *CI* confidence interval, *IV* inverse variance, *SD* standard deviation, *Std* standardised mean difference

**Fig. 7:**
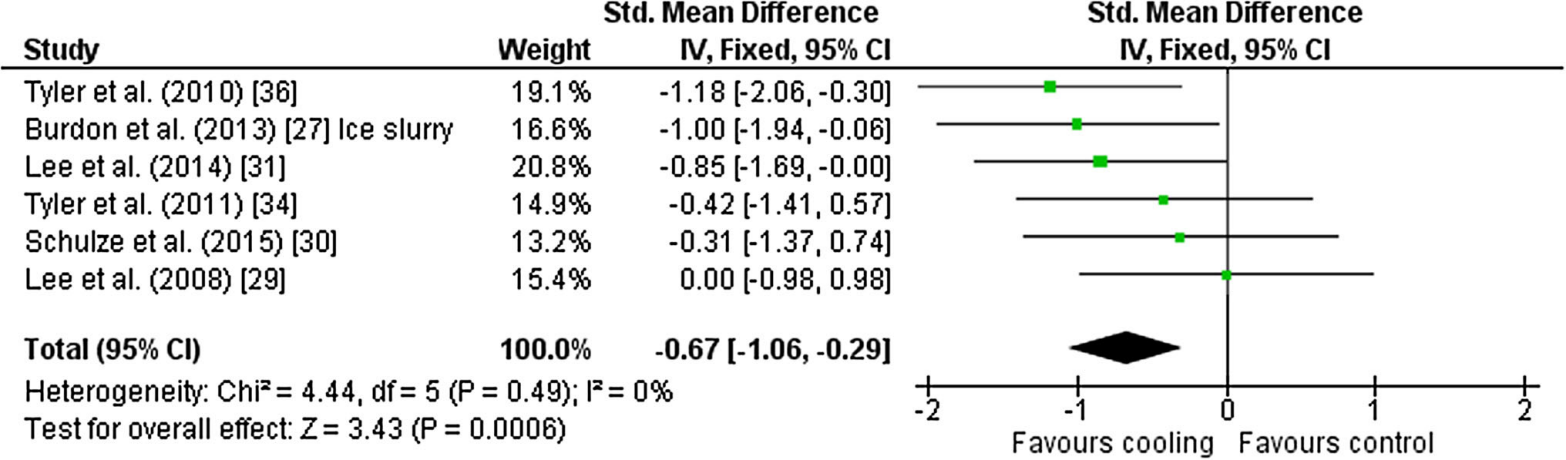
Intervention effect estimates, 95 % CIs and weighted average of the Std for thermal perception. *CI* confidence interval, *IV* inverse variance, *SD* standard deviation, *Std* standardised mean difference

Eight outcomes from seven studies [27, 31, 33, 34, 36, 38, 39] were included in the meta-analysis for mean RPE. All studies used the Borg 6–20 RPE scale. Studies used neck cooling (*n* = 4), palm cold pack, ice slurry, ice slurry mouthwash and combined forehead and neck cooling (all *n* = 1) as interventions. Cooling during exercise improved RPE [Hedges’ *g* = − 0.49 (95 % CI −0.81 to −0.17)] (Fig. 6). Six outcomes from six studies [27, 29–31, 34, 36] were included in the meta-analysis for mean thermal perception. Studies used three different scales [40–42]; one investigation did not report the scale used [31]. Studies used, neck cooling (*n* = 3), ice slurry (*n* = 2) and cold fluid (*n* = 1) as interventions. Cooling during exercise improved thermal perception [Hedges’ *g* = − 0.67 (95 % CI −1.06 to −0.29)] (Fig. 7).

### Whole Body Sweat Production

**Fig. 8:**
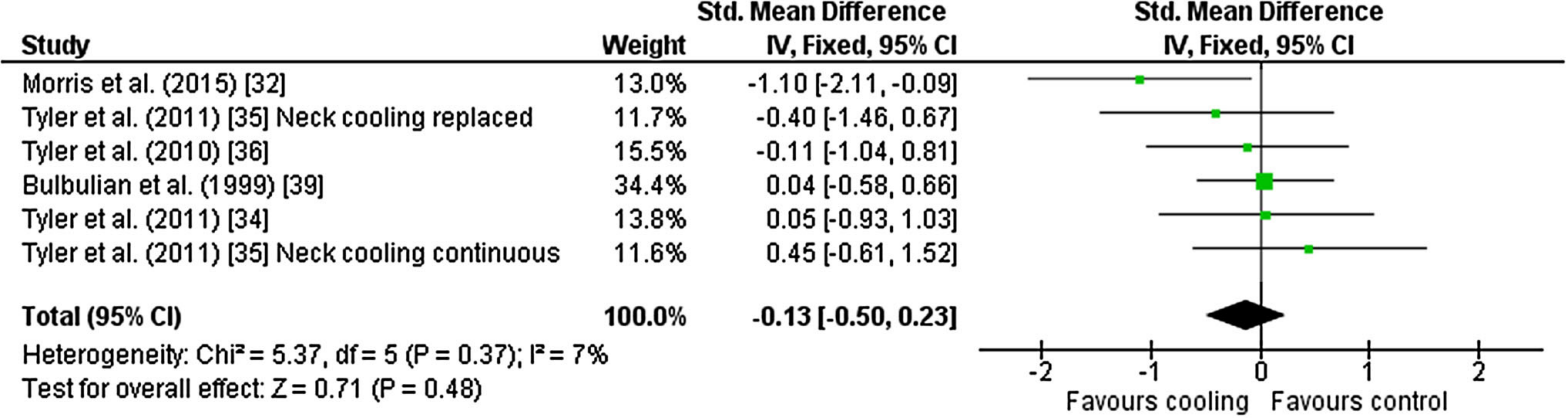
Intervention effect estimates, 95 % CIs and weighted average of the Std for whole body sweat production. *CI* confidence interval, *IV* inverse variance, *SD* standard deviation, *Std* standardised mean difference

Six outcomes from five studies [32, 34–36, 39] were included in the meta-analysis for whole body sweat production. Studies used neck cooling (*n* = 4), ice slurry and head and neck cooling as interventions (all *n* = 1). There was no effect of cooling during exercise on whole body sweat production [Hedges’ *g* = − 0.13 (95 % CI −0.50 to 0.23)] (Fig. 8).

### Exercise Duration

Four studies assessed time to exhaustion at a fixed intensity. Two studies reported mean improvements in time to exhaustion using neck cooling; however, CIs suggested no effect was observed [Hedges’ *g* = − 0.38 (95 % CI −1.37 to 0.61); *g* = − 0.58 (95 % CI −1.40 to 0.24), respectively] [31, 34]. One study used a palm cold pack [33] and reported no effect on time to exhaustion during running [Hedges’ *g* = − 0.07 (95 % CI −0.87 to 0.73)] and another used cold fluid [29] and documented no effect on time to exhaustion during cycling [Hedges’ *g* = − 0.09 (95 % CI −1.07 to 0.89)].

### Self-Paced Performance

**Fig. 9:**
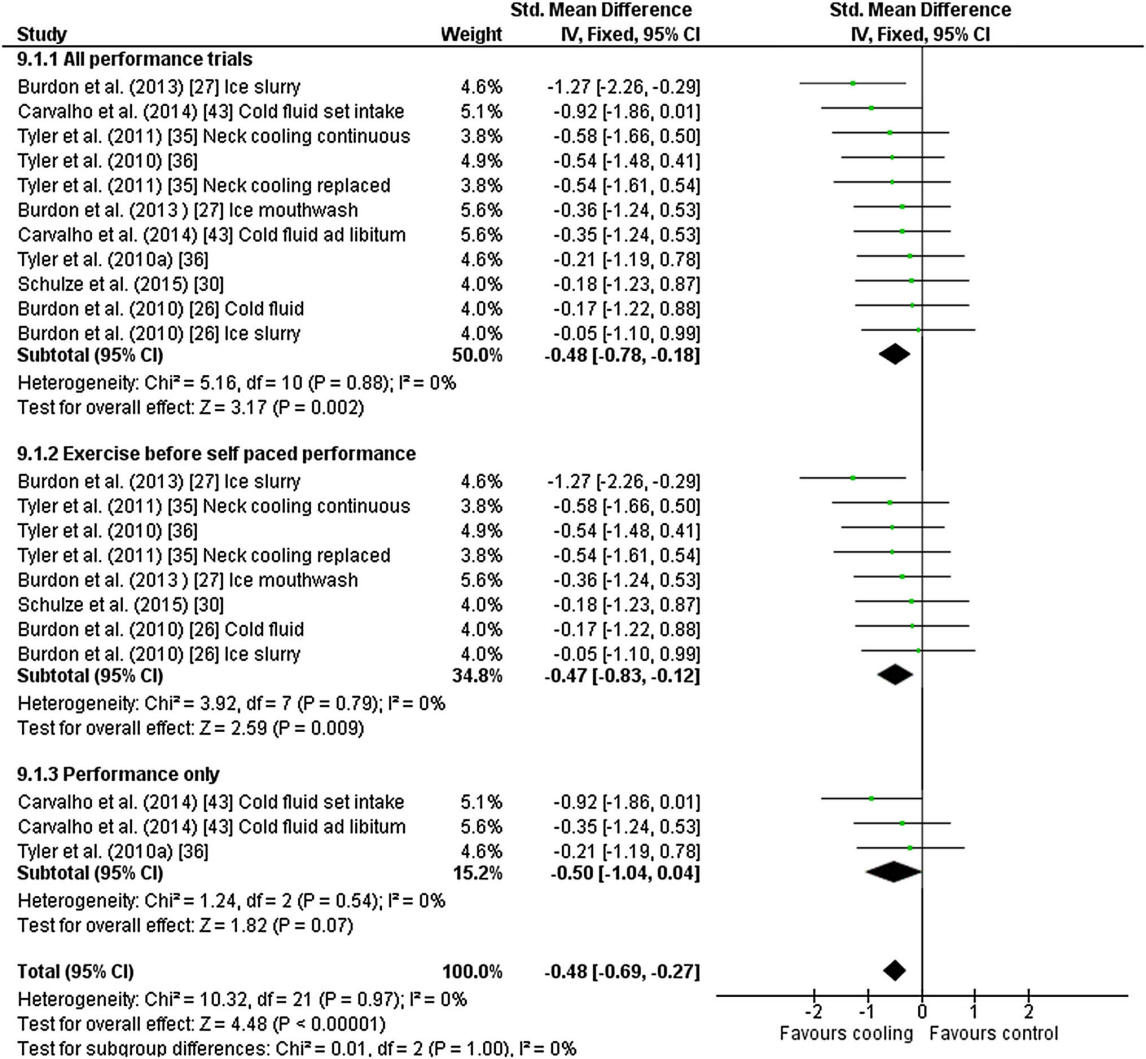
Intervention effect estimates, 95 % CIs and weighted average of the Std for self-paced performance. ‘Tyler et al. [36]’ refers to the second experiment reported in the manuscript. *CI* confidence interval, *IV* inverse variance, *SD* standard deviation, *Std* standardised mean difference

Eleven outcomes from six studies [26, 27, 30, 35, 36, 43] were included in the meta-analysis for self-paced performance. Studies used neck cooling (*n* = 4), cold fluid ingestion (*n* = 3), ice slurry (*n* = 3) and ice slurry mouthwash (*n* = 1) as interventions. There was a beneficial effect of cooling during exercise on self-paced performance [Hedges’ *g* = − 0.48 (95 % CI −0.78 to −0.18)] (Fig. 9). Sensitivity analysis demonstrated that self-paced performance was improved after fixed-intensity exercise [Hedges’ *g* = − 0.47 (95 % CI −0.83 to −0.12)] (Fig. 9), but the effect was only evident following a fixed-intensity pre-load and not for self-paced performance trials only.

## Discussion

The purpose of this review was to investigate the effects of practical cooling strategies used during continuous exercise in hot environments. Meta-analyses revealed no effect for cooling during exercise on mean core temperature, end-exercise core temperature, mean skin temperature, mean heart rate, whole body sweat production and stand-alone self-paced performance; although CIs for these estimates overlapped beneficial and detrimental effects, suggesting uncertainty in the point estimate, we interpret these findings as being unclear and that more data are required to improve confidence in the interpretation of these outcomes. Cooling during exercise, however, was beneficial for self-paced exercise performance when cooling was applied during a period of fixed-intensity exercise before the trial; RPE and thermal perception were also improved by cooling. The unclear effects on stand-alone self-paced performance and improved RPE and thermal perception oppose findings from previous meta-analyses [18, 19]. These studies found clear improvements in performance but unclear findings for perceptual responses. These contradictions are likely due to differences in study selection.

Our systematic search strategy identified 14 studies that met our inclusion criteria, whereas a previous meta-analysis included up to nine studies [19]. Tyler et al. [19] noted that some cooling methods might have limited practicality (e.g. excess mass, skin irritation and sport-rule constraints); thus we only included studies that were deemed to be practically applicable during continuous exercise. We excluded studies that used ‘face/head fan or water cooling’, ‘liquid/air cooling garments’, ‘partial limb water immersion’, ‘ice cooling vests’, and ‘fluid bolus >400 ml’; these strategies were deemed to be impractical, especially during competition. Studies utilising these methods, however, were included in the two previous meta-analyses on cooling during exercise [18, 19]. Bongers et al. [18] and Tyler et al. [19] found that ice jackets had the greatest effect on performance; Tyler et al. [19] recognised this and subsequently offered an alternative mean-weighted standardised difference estimate with the ice jacket study [44] excluded from their analysis. However, this was because the study was conducted in an uncompensable environment, not because of the unsuitability of ice jackets.

Tyler et al. [19] did not include cold water or ice slurry ingestion in their analysis; however, we chose to include these methods because they are practical methods during continuous exercise in the heat. Bongers et al. [18] included one cold water ingestion study in their analysis [45], but the comparator to the experimental trial (4 °C) was also cold fluid (19 °C) and not thermoneutral; thus it was not included in the present meta-analysis. Moreover, it is unclear whether a criterion for inclusion in the previous meta-analyses required a thermoneutral or no cooling comparator trial. Whilst we recognise that studies might be investigating ‘applied’ practices, using a thermoneutral trial as a comparator is preferable to understand the true effect of cooling during exercise, particularly the mechanisms of action, which is an important component of translational physiology [23].

To understand the mechanistic actions of cooling during exercise, we discriminated between studies that used fixed-intensity exercise and self-paced performance trials. During self-paced exercise, participants are free to choose external intensity and regulate performance, but between-trial differences in intensity make interpretations of thermophysiological data difficult. During fixed-intensity exercise, however, thermophysiological effector responses are forced [46]. Such responses (e.g. core temperature) are typically reliable [47] and enable valid comparisons between interventions. Bongers et al. [18] did not discriminate between fixed-intensity exercise or self-paced performance and it was not the aim of Tyler et al. [19] to do so, but both studies reported thermophysiological responses. This distinction between study designs is a possible explanation for the differences reported in perceptual responses between the present study and that of Tyler et al. [19]. Our analysis indicates that RPE and thermal perception are improved with cooling during fixed-intensity exercise; however, Tyler et al. [19] reported unclear findings.

We discriminated between time-to-exhaustion trials and self-paced performance trials, which the two previous meta-analyses did not. These types of test are suggested to be regulated by different mechanisms; in self-paced trials participants are free to choose external intensity, and whilst this choice might have a physiological origin, behaviour is regulated by RPE within acceptable limits for the task [2] to avoid excessive physiological strain. In fixed-intensity exercise, participants cannot freely choose external intensity and the increasing demands on cardiovascular, neuromuscular and central nervous systems as a result of metabolic heat production and heat storage [48] integrate with psychological factors to determine time to exhaustion. Therefore, these types of tests should be analysed separately to avoid confusion in interpretation. Furthermore, it is well established that time-to-exhaustion tests have a greater magnitude of test-retest error than time trials [49] and such variance might contribute to wider CIs resulting in an unclear effect. Indeed, we found unclear effects of neck cooling [31, 34], cold fluid ingestion [29] and a palm cold gel pack [33] on exercise time to exhaustion.

### Core Temperature

Bongers et al. [18] suggested that cooling during exercise might attenuate the increase in core temperature, increase heat storage capacity and improve exercise capacity based on the theory for a single terminal tissue temperature (‘critical core temperature’) for cessation of exercise. This assertion was based upon the purported effectiveness of an ‘aggressive’ cooling strategy using an ice vest [50]. Ice vests, however, are not practical for use during continuous exercise performance and the mechanistic theory (critical core temperature hypothesis) underpinning this recommendation is likely too simple to explain human behavioural thermoregulation [48, 51]. Nevertheless, we investigated this hypothesis using fixed-intensity exercise trials. None of the practical cooling methods demonstrated clear reductions in either mean or end-exercise core temperature. A possible explanation is that the enthalpy of cooling methods was insufficient or the site at which cooling was applied had limited tissue perfusion (required for effective heat transfer). This might have been the case in cold water and slurry ingestion given the documented reduction in splanchnic blood flow during exercise in the heat [52] and also for neck cooling. Continuous cooling at a site with potential for high rates of perfusion would seem to be an ideal method for attenuating increases in body temperature.

### Perceptual Responses

Flouris and Schlader [2] suggested that thermal perception is an important mediator of behavioural thermoregulation that integrates with RPE in its role as the predominant controller of the intensity of exercise. We found that RPE and thermal perception were improved by cooling during fixed-intensity exercise, and this is a key finding from our analysis. Prior to increases in core temperature, self-selected intensity of exercise is likely mediated by thermal perception and its influence on RPE, whereas when core and skin temperature are elevated, cardiovascular strain is a key RPE input [2]. Studies included in the present meta-analysis used neck cooling, ice slurry and fluid ingestion. Cooling the neck during heat exposure elicits feelings of thermal comfort at rest [53], a finding extended to two [31, 36] of three studies of neck cooling during exercise. We found an unclear effect for one study [34]; the reason for this is unknown as the neck cooling collar was the same and participants and environmental conditions were similar in all three investigations. The study, however, was designed to investigate time to exhaustion and the final core temperature was >39 °C; therefore cardiovascular strain might have been the key RPE mediator rather than thermal perception. However, it is worth noting that RPE was similar between conditions. There was also a clear beneficial effect of ice slurry ingestion on thermal perception in one study [27], but not in another [30]. These differences might be attributed to the study design, specifically, a beneficial effect of set-planned [27] rather than ad libitum [30] ingestion of slurry. Lee et al. [29] reported similar between-trial responses for thermal perception (400 ml of 10 °C fluid versus 37 °C fluid ingested at 15-min intervals), although the mean ambient temperature of 25.3 °C combined with an intensity of 50 % $\dot{V}$O_2max_ was among the least thermally stressful of included studies; indeed final core temperature was 38.11 °C, less than the mean of included studies, which was 38.48 ± 0.58 °C.

Six out of eight studies reported mean improvements for RPE. Standardised mean differences for neck cooling [34] and palm cold pack [33] were similar for experimental and comparator trials. It is unclear whether palm cooling occurred in the latter study [33] because palm skin temperature was not reported; thus absence of cooling might be an explanation for the similar between-trial RPE. Nevertheless, the weighted standardised mean difference and 95 % CI indicate that cooling during exercise has the potential to improve RPE; possibly mediated via beneficial effects of cooling on thermal perception.

### Performance

Our findings indicate that cooling during fixed-intensity exercise before a self-paced time trial improves performance (Fig. 9). Strictly, this could be considered cooling before performance, but we included these studies in our analysis because of the number of studies that have classified this type of design as cooling during exercise. These data are informative for those sports whereby ‘sub-maximal’ intensities precede an intense period of activity, such as in team road cycling. In these circumstances, tactical efforts are used to position a rider (or group) for an ‘attack’ at key points within a race. Cooling during the sub-maximal phase of the race, whereby fluid ingestion and external cooling aids are less constrained by logistics, might benefit performance in a subsequent ‘attack’. In stand-alone self-paced performance trials, there are usually observable ‘end spurts’, which prior cooling might also benefit; however, we did not investigate pacing profiles in these studies. Furthermore, we found unclear effects of cooling during stand-alone self-paced performance trials, but the three trials included in the meta-analysis all reported mean improvements; a larger sample size would improve the precision of the effect.

### Skin Temperature and Heart Rate

We observed unclear effects of cooling during exercise on mean skin temperature and heart rate during fixed-intensity exercise. Burdon et al. [28] investigated a potential link between ice slurry ingestion, decreased skin temperature and heart rate during exercise. Although Burdon et al. [28] reported a statistically significant difference (*P* < 0.05) between ice slurry and thermoneutral trials for mean skin temperature towards the end of 90 min of cycling at 62 % $\dot{V}$O_2max_ in 30 °C, our analysis found an unclear effect on mean skin temperature [Hedges’ *g* = − 0.88 (95 % CI −1.81 to 0.05)] for this study. These unclear effects were replicated across all included studies apart from Burdon et al. [26], where there was a beneficial effect of cold fluid ingestion (4 °C, ~185 ml every 10 min for 90 min) on mean skin temperature [Hedges’ *g* = − 1.40 (95 % CI −2.62 to −0.19)]. There were no beneficial effects on heart rate across the range of included studies (Fig. 5) in the present meta-analysis. This does not, however, indicate there were no beneficial effects on skin blood flow or stroke volume, as skin temperature and heart rate are only indexes of these variables in this context.

The effect of cooling during exercise on whole body sweat rate was unclear (Fig. 8), although one study [32] reported a clear decrease in whole body sweat rate after ingesting 3.2 ml·kg^−1^ of ice slurry (~240 ml) at three 15-min intervals (15, 30 and 45 min) during the first 45 min of 75 min of exercise. The authors suggested that intra-abdominal thermoreceptors integrate with the central nervous system to elicit strong thermoeffector responses at the skin surface, in particular sudomotor function. Therefore, despite an internal heat sink caused by slurry ingestion, the decreased evaporative heat loss impaired net heat loss and increased heat storage. It should be noted that this was the only study included in the meta-analysis that used ice slurry during exercise to investigate whole body sweat rate responses; more research is required to corroborate these findings.

### Study Methods and Reporting

All studies included in the present investigation were randomised cross-over trials; however, it was unclear as to how randomisation and allocation concealment occurred (Table 3). Researchers should report this information to facilitate appraisal of bias and study quality. Some studies (e.g. Tyler and Sunderland [35]) reported changes in local temperature as a result of cooling; however, most studies did not. Tyler et al. [19] recognised that such information is required to confirm whether local cooling occurred as a result of the intervention and is particularly relevant for external cooling methods. In addition, researchers should indicate the practicality of the method and report feelings of uncomfortableness, irritations, adverse effects and general appraisals from participants. This information would be helpful to scientists, coaches and athletes who are in the process of evaluating the suitability of a particular cooling method for their own specific use.

**Table 3:** Risk of bias assessment

Study	Random sequence generation	Allocation concealment	Blinding of participants and personnel	Blinding of outcome assessment	Incomplete outcome data	Selective reporting
Burdon et al. (2010) [26]	?	?	−	?	+	+
Burdon et al. (2013) [27]	?	?	−	?	+	+
Burdon et al. (2015) [28]	?	?	−	?	?	+
Lee et al. (2008) [29]	?	?	−	?	?	+
Lee et al. (2014) [31]	?	?	−	?	?	+
Minniti et al. (2011) [38]	?	?	−	?	?	+
Morris et al. (2015) [32]	?	?	−	?	?	+
Scheadler et al. (2013) [33]	?	?	−	?	+	+
Schulze et al. (2015) [30]	?	?	−	?	+	+
Tyler et al. (2010) [36] Tyler et al. (2010) [36]	?	?	−	?	?	+
Tyler and Sunderland (2011) [34]	?	?	−	?	?	+
Tyler and Sunderland (2011) [35]	?	?	−	?	?	+
Bulbulian et al. (1999) [39]	?	?	−	?	?	+
Carvalho et al. (2014) [43]	?	?	−	?	?	+

*+* Low risk of bias, *−* high risk of bias,* ?* unclear risk of bias

### Limitations

A potential limiter to the application of all cooling studies is that there is no consensus as to what constitutes a practical or impractical cooling strategy. The suitability of a particular technique is ultimately based upon complex interplay between the logistical constraints of the situation, coaching philosophy and athlete perceptions. Nevertheless, researchers have investigated strategies that might not be feasible for use during continuous exercise, a problem that has been previously identified in the scientific literature [19, 21]. In the absence of such consensus, we have excluded strategies that we deem to be impractical for use during actual competition. This might have introduced selection bias within our included studies, but we are confident that we have captured strategies that are useful for athletes, coaches and scientists. Including studies that used impractical strategies would have limited the ecological validity of the present review and thus its applied impact. We acknowledge that the data presented here are based entirely on samples from male participants. There is some evidence indicating a lower sweat rate in females compared with males [54]; however, we did not find any differences in sweat rate as a result of cooling. Nevertheless, a reduced capability for sweating and lower aerobic capacity might feed forward to thermal perception and RPE. Therefore we recommend cautious application of our findings to females and encourage future research in this area.

### Recommendations

Our analysis provides evidence that self-paced performance is improved when the cooling strategy is administered during continuous exercise before the performance trial. Ice slurry ingestion and neck cooling are the most studied practical cooling interventions and are both associated with beneficial effects on thermal perception, RPE and performance. We suggest that improvements in self-paced performance are mediated via the beneficial effects of cooling on thermal perception and RPE. This is consistent with human behavioural thermoregulatory theory [2], which states that self-selected intensity of exercise increases or decreases dependent on the magnitude of thermal or cardiovascular strain, which integrates with the predominant intensity controller, RPE. In principle, participants felt cooler during exercise and perceived the intensity of exercise to be less. Consequently, participants chose to increase external intensity, resulting in a performance improvement. This is clearly beneficial for high-performance athletes; however, it is associated with a risk of heat-related illness, particularly for novice and youth athletes, as an increased intensity causes a greater magnitude of metabolic heat production, heat storage and body temperature. This combination is of particular concern if the environment is uncompensable or the thermoregulatory responses of the participant are inadequate to equilibrate the basic heat balance equation [1].

Practitioners should also be aware relatively large volumes of ice slurry (240 ml) or cold water ingestion (>400 ml) might decrease sweat gland activity and limit the potential for evaporative heat loss, resulting in heat storage and high body temperature. This is another concern for underprepared novice and youth populations whose whole body sweat responses and evaporative heat transfer potential are likely inadequate to match that required to attain heat balance. Such a bolus, however, is associated with discomfort [32], and an ingestion of this magnitude would likely be avoided ad libitum. We are not aware of any meaningful detriment on whole body sweat rate occurring due to neck cooling; however, neck cooling is associated with the attainment of a high body temperature (Fig. 3). Indeed, none of the practical cooling methods were sufficient to attenuate an increase in body temperature. Therefore, we suggest practitioners undertake a thorough evaluation of the environment where competition or training will take place and that metabolic heat production and evaporative heat loss requirements are estimated prior to activity. Adequate body temperature, fluid balance and perceptual monitoring procedures [3, 55] should be in place, especially for highly motivated novice and youth athletes. Such an approach will improve the likelihood that appropriate cooling strategies are implemented during exercise. To date, no studies have investigated a combination of neck cooling, ice slurry and cold fluid ingestion; these strategies might have additive effects and be more beneficial than administering a single method alone. Future research should also consider exploiting sites, such as the hands, that have potential to attenuate increases in body temperature [56, 57]. In addition, opinions of coaches, athletes and support staff regarding the practicality of cooling methods should be evaluated to guide scientists towards research that has high ecological validity and sound mechanistic underpinning.

## Conclusion

We found that practical cooling strategies administered during exercise before a self-paced endurance trial improve performance in hot environments, but not by decreasing core temperature as previously thought [18]. Instead we suggest that current methods improve performance by benefiting thermal perception and RPE, resulting in greater self-selected external intensities compared with a thermoneutral or no cooling trial, thus improving endurance performance. We encourage practitioners to explore the use of cold fluid, ice slurry ingestion and neck cooling for endurance performance enhancement after examining the thermal constraints of the environment. Future research should investigate a combination of approaches to cooling during continuous exercise as well as additional sites, such as the hands, that have the potential to attenuate increases in body temperature.
